# Exploring the Potential of ChatGPT for the Summarization of Patient Medical Histories: A Pilot Study

**DOI:** 10.7759/cureus.84133

**Published:** 2025-05-14

**Authors:** Mayuko Karino, Clara So, Torahiko Jinta, Michiho Tanaka, Tomoaki Nakamura, Kohei Okafuji, Atsushi Kitamura, Yutaka Tomishima, Naoki Nishimura

**Affiliations:** 1 Department of Pulmonary Medicine, Thoracic Center, St. Luke's International Hospital, Tokyo, JPN; 2 Department of Pulmonology, St. Luke's International Hospital, Tokyo, JPN

**Keywords:** artificial intelligence, chatgpt, deep-learning, discharge summary, large language models

## Abstract

Improvements in operations through the use of artificial intelligence (AI) are expected in various fields. Chat Generative Pre-trained Transformer (ChatGPT), released in November 2022, has gained rapid popularity and is widely used; however, from a privacy perspective, research on the use of AI in medicine is limited. In this study, we compared 68 discharge summaries generated by ChatGPT based on interim summaries (hereafter, “AI summaries”) with those generated by junior residents (hereafter, “resident summaries”) and investigated the challenges and benefits of using ChatGPT. Resident summaries significantly outperformed AI summaries in quality (p = 0.011), factuality (p < 0.002), and completeness (p < 0.001) but not in readability (p* *= 0.08). For hospitalizations exceeding three months (n = 12), no significant differences were observed between resident and AI summaries on any measure. These findings suggest that AI can produce discharge summaries comparable to those of residents in complex, long-term cases. Thus, responsible use of ChatGPT, ensuring data privacy, can aid in efficient discharge summary creation.

## Introduction

Artificial intelligence (AI) has evolved considerably over the past five decades and revolutionized many industries. In the 2000s, deep learning emerged as a powerful machine-learning technique that could recognize complex patterns and exhibit self-learning capabilities. This led to significant performance gains compared to other methods [1]. Launched in November 2022, Chat Generative Pre-trained Transformer (ChatGPT) is a large AI-based multimodal model that can solve difficult problems [2]. Although the use of ChatGPT has the potential to improve the productivity of human activities, there are also concerns about ethics, potential biases, lack of originality, and cybersecurity issues [3]. In the medical field in particular, large language models may produce inaccurate content, which increases the risk of disseminating incorrect medical information and potentially causing harm to patients [4]. However, little consideration has been given to its use in the medical field due to privacy concerns such as unauthorized access to highly sensitive patient data, data breaches, and reidentification of anonymized patient information [5-7].

When a patient is discharged from the hospital, a discharge summary is created. However, summarizing a longer hospital stay becomes more challenging. In the case of long-term hospitalization, doctors at our hospital create interim summaries, including at the time of a doctor change. The use of ChatGPT to generate discharge summaries based on interim summaries has the potential to streamline the work of doctors [8]. However, research on generative AI in the medical field is lacking [9]. This study aimed to explore both the benefits and limitations of using AI for medical history documentation. To this end, we compared AI-generated summaries (hereafter, “AI summaries”) with those generated by junior residents (hereafter, “resident summaries”). In addition, we defined cases with hospital stays exceeding three months as long-term cases and focused our analysis on comparing AI and resident summaries for these cases.

## Materials and methods

We conducted a cross-sectional study of patients admitted to the Department of Pulmonary Medicine at St. Luke's International Hospital in Japan who were discharged between April 2022 and March 2023. The study was approved by the Institutional Review Board of St. Luke’s International Hospital (review board number: 23-J008, October 2, 2023). Consent for the use of clinical data for research purposes was obtained at the time of hospital admission, in accordance with the hospital’s privacy policy, the Ethical Guidelines for Medical and Health Research Involving Human Subjects, and all other applicable regulations and guidelines. Patients who stayed for more than one month with interim summaries were included, as discharge summaries are usually created every month. Patients without interim summaries were excluded. The interim summary includes sections such as current medical history, hospital course, medications, and problem list, and is updated by physicians in our hospital approximately every month.

After confirming that no personally identifiable information as defined by the Personal Information Protection Act was included, such as the name of place and hospital, the “hospital course” section from the interim summary was entered into GPT-4 with the first instructions: “Please summarize the following information to create a discharge summary.” If there were multiple interim summaries, the last one created was used. Subsequently, instructions were given in a dialogue format in Japanese to generate AI summaries with style changes. The following instructions were used in the order listed: “Please summarize the following information to create a discharge summary.” “Write in complete sentences.” “Do not use honorifics.” “Do not use abbreviations.” “Be concise.” The AI summaries included summaries of image data such as blood test data, biochemistry, and radiological images.

**Table 1:** Definition of evaluation items

	Evaluation item	Definition
1	Quality	Concise and well-organized as a discharge summary. The patient's care plan for other doctors is easy to understand.
2	Readability	The text is suitable for a clinical doctor. It is intended to be read by another doctor. Abbreviations and medical terms are used in a way that can be understood by other doctors reading it.
3	Factuality	All information is medically consistent.
4	Completeness	Contains descriptions of relevant events that occurred during the hospitalization, ensuring that no important information is missing for the next doctor to decide on the care plan.

To prevent expectation bias, where evaluators assume summaries written by residents are superior to AI-generated ones, and confirmation bias, where evaluators actively seek errors in AI-generated expressions, the AI summaries and resident summaries were blinded, and three specialists in respiratory internal medicine assessed them based on four criteria: quality, readability, medical factuality (hereafter, “factuality”), and completeness as a summary of the medical history (hereafter, “completeness”) (Table 1). The evaluation criteria were explained by the author to the three evaluators simultaneously in advance, and a consensus was reached. A standard 10-point Likert scale, commonly used in medical research for subjective assessments [10,11], was employed as the scoring platform in this study. Each physician rated all summaries on a 1-10 Likert scale, where 1 indicated poor and 10 indicated excellent, with the criteria for each metric listed in Table 1. Based on a previous study [12], to standardize the criteria for clinical validity among the three evaluators, we set a cutoff score of 7 for clinical validity. A paired t-test was performed at a 5% significance level.

## Results

**Figure 1:**
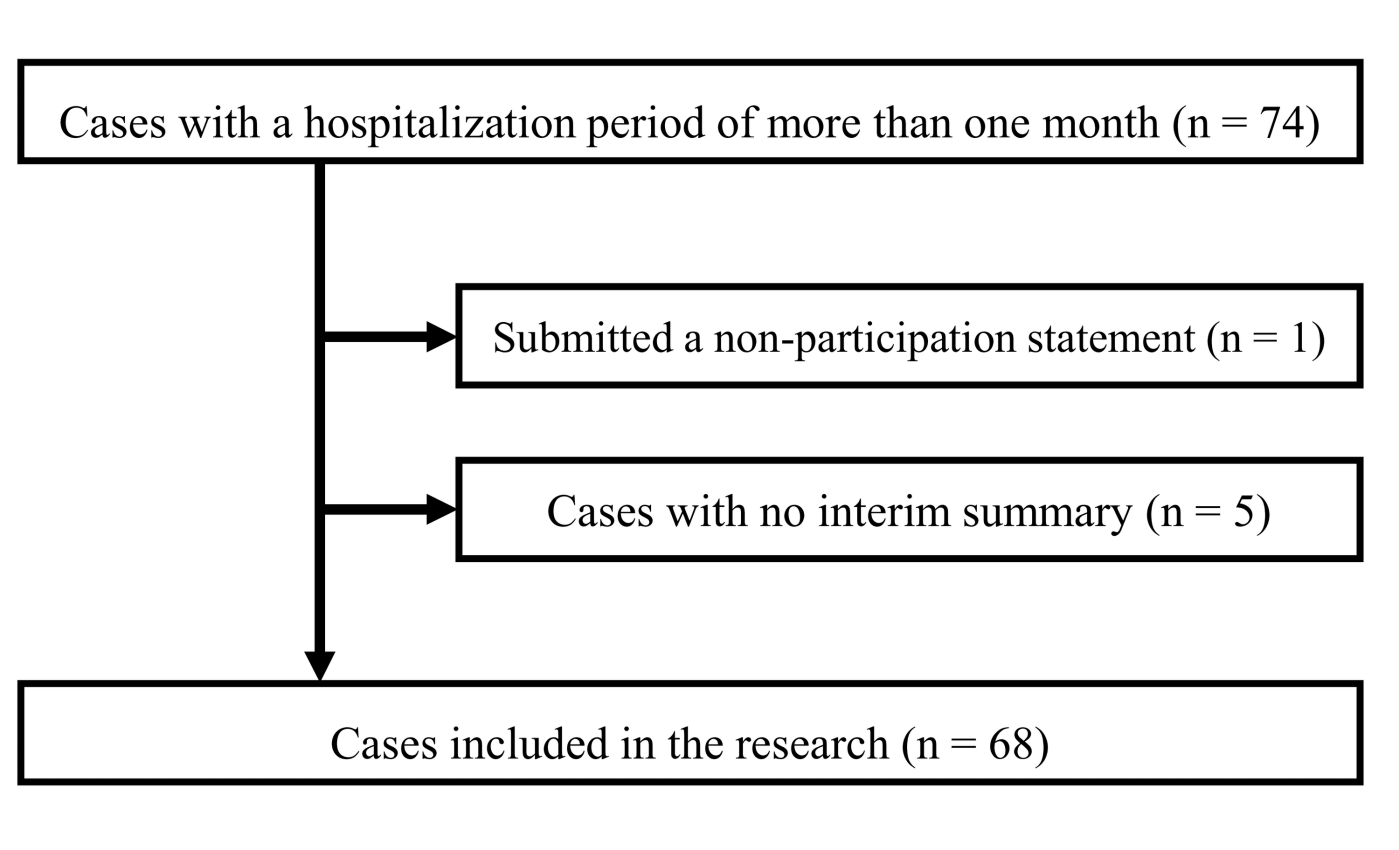
Flowchart of cases

During the study period, 74 patients were hospitalized for more than one month. Six patients were excluded: five with no interim summary and one who refused to participate. Thus, 68 patients were finally included in this study (Figure 1).

The baseline characteristics and disease details are summarized in Table 2. The median (range) age was 79 years (37-98), and 39 patients (57.4%) were male. The most common diagnoses on admission were aspiration pneumonia (24 cases (35.3%)), followed by interstitial pneumonia (13 cases (19.2%)).

**Table 2:** Baseline characteristics and disease details (n = 68)

Characteristic	Value
Age (years), median (range)	79 (37-98)
Sex, n (%)	
Male	39 (57.4)
Female	29 (42.6)
Diagnosis, n (%)	
Aspiration pneumonia	24 (35.3)
Interstitial pneumonia	13 (19.2)
Pneumonia	16 (23.5)
Lung cancer	6 (8.8)
Empyema	3 (4.4)
Nontuberculous mycobacterial infection	3 (4.4)
Others	3 (4.4)

**Figure 2:**
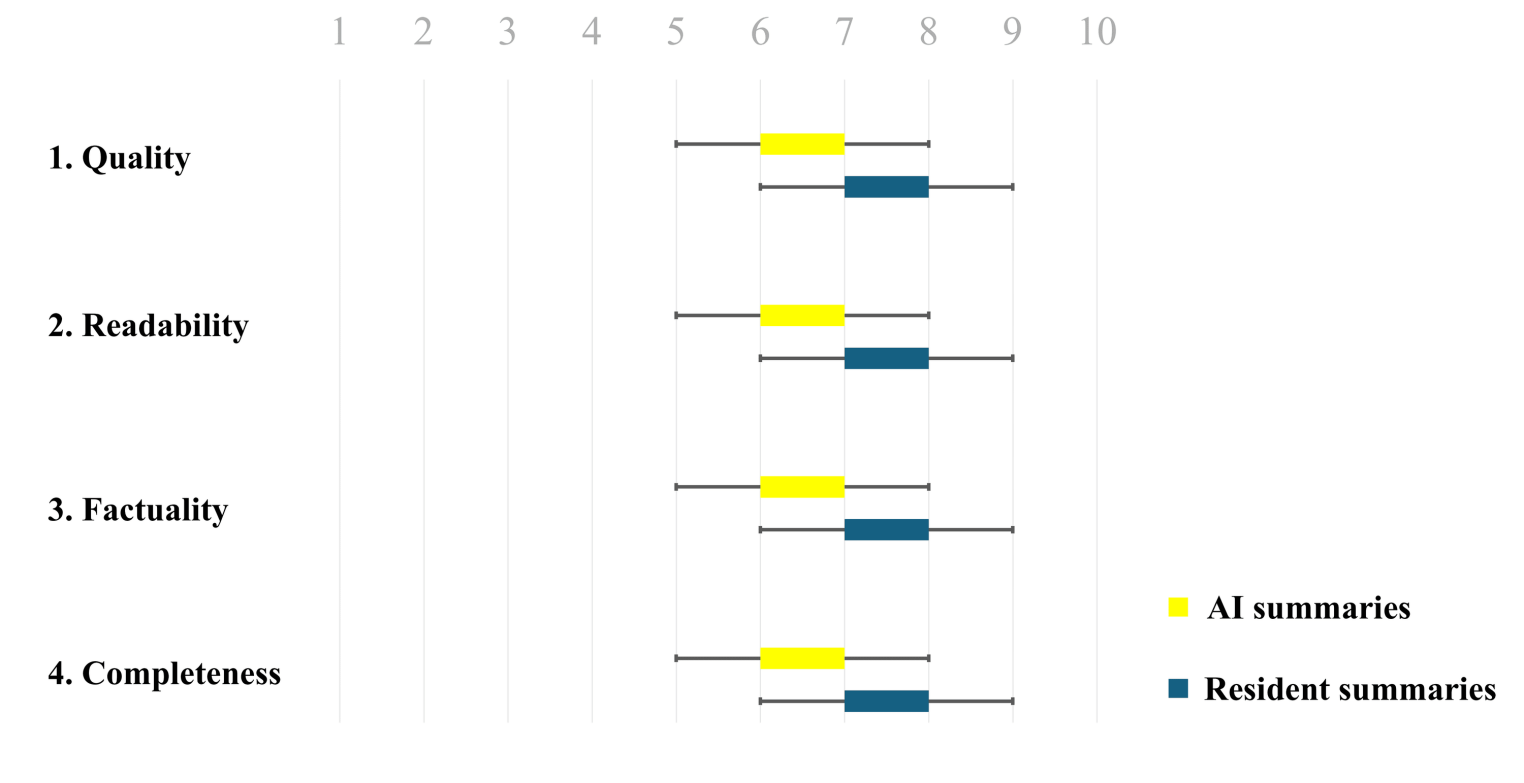
Box plot for the Likert scores for quality, readability, factuality, and completeness of the AI and resident summaries by the physician reviewers

The resident summaries scored significantly higher than did AI summaries in terms of quality, factuality, and completeness (quality: 7.16 vs. 6.80, p = 0.011; factuality: 7.21 vs. 6.77, p < 0.002; completeness: 7.24 vs. 6.56, p < 0.001), but there was no significant difference in the readability (7.09 vs. 6.8, p = 0.08) (Table 3, Figure 2).

**Table 3:** Mean and SD of the ratings for quality, readability, factuality, and completeness of the AI and resident summaries

	Mean (±SD)		p-value
	AI	Resident	
Quality	6.80 (±0.87)	7.16 (±1.00)	0.011
Readability	6.85 (±0.90)	7.09 (±0.98)	0.077
Factuality	6.77 (±1.00)	7.21 (±0.93)	0.002
Completeness	6.56 (±1.01)	7.24 (±1.00)	< 0.001

**Figure 3:**
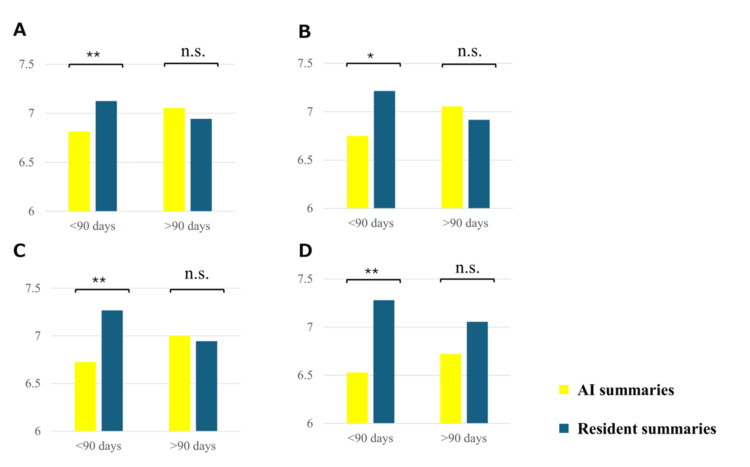
Bar graph comparing scores between the AI summaries and resident summaries for cases with a hospital stay of 90 days or more. (A) Quality; (B) readability; (C) factuality; (D) completeness * p < 0.05, ** p < 0.01

Next, cases were divided into those with a hospitalization period of more than 90 days and those with a hospitalization period of less than 90 days. The median (range) age of cases with a hospitalization period of more than 90 days was 71 years (29-82), and eight patients (66.6%) were admitted to the intensive care unit during hospitalization, and seven patients (58.3%) required mechanical ventilation. Wilcoxon rank-sum tests were performed for each of the four criteria. The resident summaries scored higher than did AI summaries in terms of completeness although there was no significant difference found between AI and resident summaries for all four items for cases with a hospital stay longer than 90 days (quality: 7.06 vs. 6.92, p = 0.95; readability: 7.06 vs. 6.94, p = 0.82; factuality: 7.00 vs. 6.94, p = 1.00; completeness: 6.72 vs. 7.06, p = 0.18) (Table 4, Figure 3).

**Table 4:** Mean and SD of the ratings for quality, readability, factuality, and completeness of the AI and resident summaries for cases with a hospital stay of 90 days or more

	Mean (±SD)		p-value
	AI	Resident	
Quality	7.06 (±1.12)	6.92 (±0.58)	0.95
Readability	7.06 (±0.73)	6.94 (±0.52)	0.82
Factuality	7.00 (±1.14)	6.94 (±0.57)	1.00
Completeness	6.72 (±1.08)	7.06 (±0.49)	0.18

## Discussion

In this study, the usefulness and challenges of AI in the medical field were demonstrated by comparing AI summaries with resident summaries. In terms of quality, factuality, and completeness, resident physician summaries significantly outperformed AI summaries. However, focusing on length of stay, there was no significant difference between AI and resident summaries in cases of long-term hospitalization lasting 90 days or more. Although our study focused on respiratory diseases as a pilot, the approach could theoretically be applied to other medical fields.

Clough et al. compared discharge summaries by AI and clinicians using simulated patient vignettes, which contained pertinent information such as diagnosis and reason for admission, treatments received, and any discharge recommendations, with no specific duration of stay defined. They reported that all summaries produced by AI were of sufficient quality, demonstrating that AI can produce discharge summaries at a level comparable to those by junior doctors [13]. Similarly, Scott et al. reported that AI-generated text summaries could be as accurate as those written by clinicians, which would be more efficient for clinicians to accurately identify key information about the patient's medical history [14]. However, this study showed the overall superiority of resident physician summaries over AI summaries, particularly in terms of completeness, suggesting that the AI summaries were incomplete, possibly because they could only be written up to the point of producing interim summaries.

There was no significant difference between AI and resident summaries in cases of long-term hospitalization lasting 90 days or more. While some long-term hospitalized patients were relatively young, many had pre-existing conditions, such as congenital disorders or hematologic diseases. Among elderly patients, repeated episodes of aspiration pneumonia and urinary tract infections were frequently observed. Many of these cases were severe and required multidisciplinary treatment. The long-term hospitalization group had more clinically complex and serious patients with more information in the interim summaries. The absence of a significant difference in performance between AI summaries and resident summaries in this group may be explained by the difficulty residents face in synthesizing large volumes of data.

In addition, the time required to summarize was only a few seconds. In our study, manually instructing AI to generate summaries may not have effectively reduced the workload. However, this approach highlighted the potential for workload reduction in the future when AI can autonomously collect patient information for summary creation. In cases of short-term hospitalization, interim summaries were unavailable, so the usefulness of ChatGPT could not be demonstrated in this study. However, if clinical information can be automatically extracted from medical records, it is possible to generate efficient and comprehensive discharge summaries. Moving forward, it is essential to prioritize data privacy in the use of AI in medical contexts. This could involve restricting AI's access to electronic medical records or ensuring its operation within secure systems that adhere to established guidelines [15,16].

Although AI effectively summarized medical histories, it also struggled to assess the severity of complex cases with multiple coexisting conditions, lacking the attending physician's judgment. Clough et al. noted that while automation allows AI to show less variability than human documentation, they also pointed out that AI may struggle to capture and effectively communicate individual nuances [13]. Additionally, clear errors surfaced, such as using medical terms absent from the actual summaries. These limitations highlight that despite advancements in AI for medical settings, doctor confirmation remains crucial. Previous research has also discussed the potential of using ChatGPT to generate discharge summaries, while they also highlighted that a minor error could greatly affect patient care, which is consistent with our view [8,17]. Integrating domain-specific medical knowledge bases with general-purpose language models could significantly improve the clinical accuracy, factual consistency, and contextual relevance of AI-generated summaries, addressing these challenges. Incorporating specialized medical terminology and up-to-date clinical guidelines would allow the AI to better align with the specific requirements of the healthcare setting. This would reduce the likelihood of errors and enhance the AI's reliability for clinical use.

 In this study, Japanese was used as the language for interacting with ChatGPT-4. It is important to note that ChatGPT-4's response quality varies with language. Although ChatGPT-4's performance in Japanese is not as robust as it is in English, it performs much better than ChatGPT-3.5 did in English [18]. The development of multilingual models that minimize performance disparities across languages is essential to ensure consistent and high-quality responses. Enhanced multilingual capabilities would enable healthcare professionals to generate documentation and provide explanations accurately and efficiently in their native languages, ultimately contributing to improved overall quality of patient care. Additionally, instructions were given to ChatGPT in a conversational format, with multiple instructions provided sequentially. When all instructions were given simultaneously, not all conditions were consistently met, resulting in varied responses based on the method of instruction delivery. Duong et al. also found that ChatGPT frequently provided different answers when asked the same question multiple times [19].

This study has two limitations. First, owing to constraints in automatically accessing electronic medical records for privacy reasons, the program relied solely on interim summaries. Given that not all hospitals generate interim summaries, and they may not capture all relevant information, in the future, research should explore AI-generated summaries under more automated and comprehensive data collection protocols, while adhering to regulatory standards. Hartman et al. attempted to produce discharge summaries by automatically collecting information from medical records, laboratory data, and vital signs and reported that 62% of the automated summaries met standards of clinical validity, demonstrating the potential of automated summaries [9]. Second, the comparison between the AI and resident summaries was not truly blinded. Expressions suggestive of ChatGPT may have alerted raters to whether the summary was generated by AI or a resident physician, potentially influencing the rating. Such recognition could have introduced expectation bias, leading evaluators to unconsciously assign scores based on assumptions, such as the belief that summaries written by humans are inherently superior.

## Conclusions

This study found that summaries generated by junior residents outperformed those produced by AI in terms of quality, accuracy, and completeness. However, in complex clinical scenarios involving long-term hospitalization, AI demonstrated comparable performance in summarizing the length of stay. These findings highlight the potential of AI to support the efficient processing and summarization of extensive medical data. With continued advancements in domain-specific medical AI and automated data extraction from electronic health records, the utility of AI in clinical practice is expected to increase. Nevertheless, it remains essential for clinicians to review the summaries to assess the severity of complex cases and correct any errors that may have occurred. The integration of AI into clinical practice also raises numerous ethical and privacy concerns, necessitating the development of robust guidelines and ethical standards that can adapt to the rapidly evolving landscape of medical AI technologies.
